# Rota crítica de mulheres em situação de violência: revisão integrativa

**DOI:** 10.26633/RPSP.2019.34

**Published:** 2019-03-27

**Authors:** Daniella Yamada Baragatti, Ana Carine Arruda Rolim, Cristiane Pereira de Castro, Márcio Cristiano de Melo, Eliete Maria Silva

**Affiliations:** 1 Faculdade de Enfermagem (FENF/UNICAMP) Faculdade de Enfermagem (FENF/UNICAMP) Universidade Estadual de Campinas CampinasSP Brasil Universidade Estadual de Campinas, Faculdade de Enfermagem (FENF/UNICAMP), Campinas (SP), Brasil.; 2 Escola Multicampi de Ciências Médicas (EMCM/UFRN) Escola Multicampi de Ciências Médicas (EMCM/UFRN) Universidade Federal do Rio Grande do Norte CaicóRN Brasil Universidade Federal do Rio Grande do Norte, Escola Multicampi de Ciências Médicas (EMCM/UFRN), Caicó (RN), Brasil.; 3 Faculdade de Saúde Pública (FSP/USP) Faculdade de Saúde Pública (FSP/USP) Universidade de São Paulo São PauloSP Brasil Universidade de São Paulo, Faculdade de Saúde Pública (FSP/USP), São Paulo (SP), Brasil.; 4 Faculdade de Ciências Médicas (FCM/UNICAMP) Faculdade de Ciências Médicas (FCM/UNICAMP) Universidade Estadual de Campinas CampinasSP Brasil Universidade Estadual de Campinas, Faculdade de Ciências Médicas (FCM/UNICAMP), Campinas (SP), Brasil.

**Keywords:** Método do caminho crítico, comportamento de busca de ajuda, violência contra a mulher, maus-tratos conjugais, violência doméstica, Critical pathways, help-seeking behavior, violence against women, spouse abuse, domestic violence, Vías clínicas, conducta de búsqueda de ayuda, violencia contra la mujer, maltrato conyugal, violencia doméstica

## Abstract

**Objetivo.:**

Conhecer a rota crítica de mulheres em situação de violência doméstica no mundo em sua busca por ajuda.

**Métodos.:**

Revisão integrativa, com busca realizada nas bases de dados LILACS, MEDLINE via PubMed, EMBASE, Scopus e Web of Science. Não houve restrição quanto ao ano inicial das publicações, porém estabeleceu-se 2017 como ano final. Foram incluídos artigos com disponibilidade do texto integral *on-line*, publicados em português, inglês ou espanhol, que tratassem do tema da pesquisa e respondessem à pergunta norteadora (“Qual a rota crítica de mulheres em situação de violência doméstica?”).

**Resultados.:**

Foram incluídos 38 artigos, publicados de 2001 a 2017. Entre os fatores impulsores da busca por ajuda identificaram-se empoderamento econômico e alta escolaridade, severidade da violência e presença de serviços de apoio estruturados e qualificados. Como inibidores identificaram-se o fato de a mulher ser imigrante, a existência de normas culturais de gênero, sentimentos de culpa, medo e vergonha, falta de confiança e pouco conhecimento e/ou disponibilidade limitada de serviços de apoio formal. Filhos, apoio da família e comunidade podem ser fatores inibidores ou impulsores da busca por ajuda. Os tipos de ajuda formal mais procurados são a polícia e os serviços de saúde, enquanto a família, a comunidade e as lideranças religiosas se configuram como apoios informais.

**Conclusão.:**

A rota crítica das mulheres em situação de violência doméstica no mundo existe de maneira formal e informal. Portanto, é preciso trabalhar questões socioculturais, comunitárias e familiares para incentivar a mulher a romper com a situação de violência, incluindo a busca pela rede de apoio formal qualificada.

## Abstract (en)

**Objective.:**

To identify the critical pathway taken by women facing domestic violence in the world in their search for help.

**Method.:**

An integrative review was performed. LILACS, MEDLINE/ PubMed, EMBASE, Scopus, and Web of Science databases were searched until year 2017. The following were inclusion criteria: full text available online, Portuguese, Spanish, or English as language of publication, focus on the theme of interest, and answering the guiding question (Which critical path is taken by women facing domestic violence?).

**Results.:**

Thirty-eight articles published from 2001 to 2017 were included. Factors driving the search for help included economic empowerment and increased schooling, severity of the violence, and presence of structured and qualified support services. Factors inhibiting the search for help were immigrant status, cultural gender norms, feelings of guilt, fear, and shame, lack of confidence and little knowledge and/or limited availability of formal support services. Children as well as family and community support may function as both inhibitors or drivers of the search for help. The types of formal help most often sought are police and health care services, whereas family, community, and religious leadership provide informal support.

**Conclusion.:**

The critical pathway of women facing domestic violence in the world includes both formal and informal elements. Therefore, it is necessary to address sociocultural, community, and family issues so as to encourage women to break free from the violent environment and seek qualified formal support networks.

## RESUMEN

**Objetivo.:**

Conocer la ruta crítica que recorren las mujeres en situación de violencia doméstica en el mundo en su búsqueda de ayuda.

**Métodos.:**

Revisión integradora, con una búsqueda realizada en las bases de datos LILACS, MEDLINE vía PubMed, BASE, Scopus y Web of Science. No hubo restricción en cuanto al año inicial de las publicaciones, pero se estableció el 2017 como el año final. Se incluyeron artículos de texto completo disponible en línea, publicados en español, inglés o portugués, que trataran del tema de investigación y respondieran a la pregunta orientadora (¿Cuál es la ruta crítica que recorren las mujeres en situación de violencia doméstica?).

**Resultados.:**

Se incluyeron 38 artículos publicados desde el 2001 hasta el 2017. Entre los factores impulsores de la búsqueda de ayuda se encontraron el empoderamiento económico y el alto grado de escolaridad, la gravedad de la violencia y la existencia de servicios de apoyo estructurados y calificados. Como factores inhibidores se encontraron el hecho de que la mujer fuera inmigrante, la existencia de normas culturales de género, los sentimientos de culpa, miedo y vergüenza, la falta de confianza y el escaso conocimiento o la poca disponibilidad de servicios de apoyo formal. Los hijos y el apoyo de la familia y la comunidad pueden ser factores inhibidores o impulsores de la búsqueda de ayuda. Los tipos de ayuda formal más buscados son la policía y los servicios de salud, mientras que la familia, la comunidad y las autoridades religiosas se configuran como formas de apoyo informal.

**Conclusión.:**

La ruta crítica que recorren las mujeres en situación de violencia doméstica en el mundo comprende la búsqueda de ayuda formal e informal. Por lo tanto, es preciso trabajar en los aspectos socioculturales, comunitarios y familiares para incentivar a las mujeres a desvincularse de la situación de violencia, lo cual incluye la búsqueda de una red de apoyo formal calificada.

Várias são as formas de expressar a “violência contra as mulheres”, estando entre as mais conhecidas a violência por parceiro íntimo, o estupro, a mutilação genital feminina, o tráfico de mulheres e o feminicídio (1-7). Esse tipo de violência deixou de ser um fato isolado em sociedades distintas e tornou-se um problema de saúde pública de proporções epidêmicas (7). Dados da Organização Mundial da Saúde (OMS) (7) revelam que aproximadamente 30% das mulheres já sofreram violência física e/ou sexual, cometida principalmente pelo parceiro íntimo. Dessas, 42% apresentaram lesões. Os parceiros homens são responsáveis por 38% dos feminicídios.

Um estudo realizado em 10 países latino-americanos pela Organização Pan-Americana da Saúde (OPAS) investigou a sequência de ações e decisões tomadas pelas mulheres violentadas em sua busca por ajuda, definindo tais escolhas como “rota crítica”. O estudo mostrou que as mulheres de fato procuram auxílio para lidar com o que vivenciam. Essa busca começa quando as mulheres rompem o silêncio e contam a uma pessoa próxima, com o intuito de melhorar sua situação. O rompimento é causado por razões íntimas que impulsionam as mulheres a realizarem ações relacionadas à rota crítica, bem como pela necessidade de obter respostas e orientação quanto a condutas seguras. A demanda por respostas e orientações, por sua vez, tem impacto nos fatores que fazem com que as mulheres considerem como habitual a violência que sofrem (8). O itinerário na rota crítica não é linear, podendo passar por diversos setores, como, por exemplo, saúde, sistema judiciário, instituições policiais, escolas, comunidade e organizações não governamentais (ONG), entre outros (8). Profissionais desses setores possuem percepções distintas sobre as mulheres e a situação de violência: profissionais de saúde e assistência social identificam as mulheres como vítimas, enquanto que os da justiça e segurança pública as percebem, muitas vezes, com base nos estereótipos da sociedade. O desconhecimento dos profissionais acerca dos serviços que atendem essas mulheres pode fazer com que sejam encaminhadas para instâncias onde não receberão o apoio necessário (9).

Tendo em vista a magnitude da violência contra a mulher e a hipótese de que as mulheres em situação de violência buscam apoio, o objetivo deste trabalho foi conhecer a rota crítica da busca de ajuda das mulheres em situação de violência doméstica no mundo.

## MATERIAIS E MÉTODOS

Foi realizada uma revisão integrativa da literatura (10) a partir da seguinte pergunta norteadora: “Qual a rota crítica de mulheres em situação de violência doméstica?”. A elaboração da estratégia de busca e avaliação dos estudos baseou-se na estratégia PICO, sendo definida a população como as mulheres em situação de violência; a intervenção como fatores impulsores ou inibidores da rota crítica e os locais onde as mulheres buscaram ajuda; a comparação deu-se entre mulheres em situação de violência em diferentes países; e o desfecho foi analisado em termos de mulheres que buscaram ajuda e obtiveram alguma forma de auxílio.

A pesquisa foi realizada em abril de 2018, por meio de consulta às bases bibliográficas Literatura Latino-Americana e do Caribe em Ciências da Saúde (LILACS) (http://lilacs.bvsalud.org), MEDLINE via PubMed (https://www.ncbi.nlm.nih.gov/pmc/), EMBASE (https://www.elsevier.com), Scopus (https://www.scopus.com) e Web of Science (https://www.scopus.com). Para a busca, foram utilizados Descritores em Ciências da Saúde (DeCS) ou Medical Subject Headings (MeSH). Em inglês, foram: “Critical Path Method” (DeCS), “Critical Pathways” (DeCS e MeSH), “Help-seeking Behavior” (DeCS e MeSH), “Violence Against Women” (DeCS), “Spouse Abuse” (DeCS e MeSH), “Battered Women” (DeCS e MeSH) e “Domestic Violence” (DeCS e MeSH).

Utilizou-se a expressão booleana AND, cruzando-se sempre um dentre os três primeiros descritores com um dentre os quatro últimos. A pesquisa foi realizada em inglês em todas as bases de dados. Somente nas bases de dados LILACS e MEDLINE, a pesquisa foi feita também em português e espanhol, já que essas bases apresentam resultados de artigos publicados nesses idiomas (“Método do caminho crítico”, “Procedimentos clínicos”, “Comportamento de busca de ajuda”, “Violência contra a mulher”, “Maus tratos conjugais”, “Mulheres agredidas” e “Violência doméstica”; “Método de la ruta crítica”, “Vías clínicas”, “Conducta de búsqueda de ayuda”, “Violencia contra la mujer”, “Maltrato conyugal”, “Mujeres maltratadas” e “Violencia doméstica”*)*. Para a busca na EMBASE utilizou-se a lista de vocabulários próprios dessa base: “Clinical pathway”, “Help seeking behavior”, “Violence against women”, “Partner violence”, “Battered women” e “Domestic violence women”.

Não houve restrição quanto ao ano inicial das publicações, porém estabeleceu-se 2017 como ano final. Foram incluídos artigos com disponibilidade do texto integral *on-line*, publicados em português, inglês ou espanhol, que tratassem do tema da pesquisa e respondessem à pergunta norteadora. Excluíram-se: artigos incompletos com acesso apenas ao resumo, artigos que tratassem de violência em outras populações, teses e dissertações, cartas ao editor, revisões de literatura e artigos que não respondiam à pergunta norteadora.

Visando à sistematização, os autores desenvolveram um instrumento de coleta de dados referentes a autoria, título do artigo, periódico, país de origem do estudo, ano, idioma, objetivo, delineamento e referencial teórico, participantes e principais resultados relativos à rota crítica. O material foi analisado através de leitura crítica, que permitiu comparar e agrupar os artigos, definindo-se três categorias temáticas.

Após a exclusão das duplicações, títulos e resumos foram lidos por dois avaliadores separadamente (DYB, MC). Caso fossem considerados adequados ao tema da revisão, os artigos eram lidos na íntegra. Após a leitura dupla dos artigos, eventuais discordâncias quanto à inclusão ou exclusão foram resolvidas por um terceiro avaliador (EMS). Os dados foram analisados por DYB, ACAR, CPE e MCM.

## RESULTADOS

**FIGURA 1:**
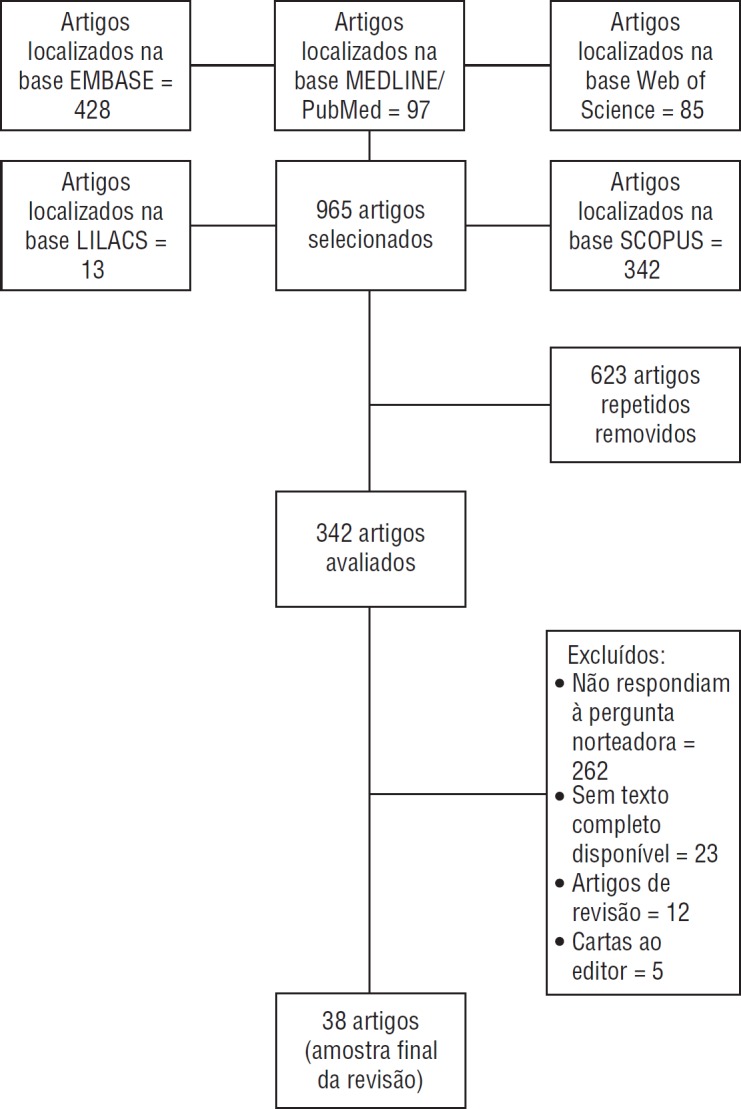
Fluxograma da seleção dos estudos que compuseram a revisão integrativa sobre busca de ajuda por mulheres que sofreram violência, 2001 a 2017

A busca nos bancos de dados revelou 965 artigos, dos quais 38 artigos compuseram o corpus (figura 1). Os principais resultados obtidos quanto à rota crítica aparecem na tabela 1 (11-48).

Identificaram-se 20 pesquisas no continente americano, cinco na África, nove na Ásia e três na Europa. Em relação à língua de publicação, 35 artigos são em inglês e três em português. O artigo mais antigo disponível é de 2001. Foram selecionados seis artigos no ano de 2009, cinco nos anos de 2017, 2015 e 2011, quatro em 2013, três em 2014 e 2012, dois em 2016 e 2003 e um artigo nos anos de 2010, 2008, 2006 e 2001.

A análise do conjunto de artigos alinhado ao objetivo deste estudo revelou três categorias temáticas, detalhadas abaixo.

### Fatores impulsores da rota crítica

Empoderamento econômico e alta escolaridade são protetores de violência por parceiro íntimo em estudos realizados nos Estados Unidos, Índia e Tanzânia (15, 23, 40, 45, 46). Existe associação entre escolaridade e procura por ajuda formal, encontrada em artigos do continente americano (13, 15, 18). Destaca-se que esses fatores de proteção não foram citados nos estudos de países como Canadá e Espanha. Estudos na Tanzânia apontam o início de um movimento onde as mulheres inserem-se nas atividades econômicas e sociais (40, 48).

Os filhos podem ser motivação para busca de ajuda ou podem representar uma dificuldade para deixar a situação de violência. Como fatores impulsores, as mulheres buscam ajuda quando enxergam a relação entre a violência vivenciada e o sofrimento dos filhos (24), para mantê-los a salvo (43), porque as crianças testemunharam situações de violência (29) ou porque as crianças incentivam a mãe a buscar ajuda (36). Em contrapartida, permanecem na situação de violência por medo de não conseguir sustentar os filhos (16), para preservar a família (17, 32), para evitar que os filhos sofram (12), por medo de perdê-los (45), porque as crianças são muito novas (39) e por acreditar que os filhos terão um futuro melhor caso permaneça com o companheiro (34).

A busca por ajuda formal cresce com a gravidade, severidade (11, 13, 15-17, 22, 24, 29, 31, 32, 38, 45, 48) e frequência da violência (11, 13, 15, 31, 32, 38, 46), possivelmente porque as mulheres precisavam de amparo jurídico ou financeiro, além de habitação, transporte, cuidados com os filhos (22) e cuidados físicos e de saúde devido a lesões físicas severas (15, 31, 45), ou porque a violência tornou-se insuportável, com ameaças de morte (13, 17, 29, 38). Outros fatores citados foram o apoio da família e comunidade (13, 24, 26, 29, 41, 43) e a presença de serviços de apoio estruturados e qualificados (13, 21, 27, 43).

### Fatores inibidores da rota crítica

Vários estudos trataram da busca de ajuda por mulheres imigrantes, sendo a imigração um motivo inibidor da busca por ajuda. No Canadá, a dificuldade de buscar ajuda pelo *status* de imigrante foi relatada por imigrantes asiáticas ou de outras origens (16, 17); nos Estados Unidos, por mexicanas, vietnamitas e asiáticas (18, 36, 42), e na Espanha, por marroquinas, romenas e equatorianas (47). As dificuldades citadas por essas mulheres na busca de ajuda são perda do apoio e isolamento social (16, 17, 42, 47), leis e políticas de imigração que, apesar de avanços, envolvem barreiras sistêmicas e estruturais (16-18, 42, 47) e desconhecimento da língua do país em que estão (16-18, 42). Destaca-se que as imigrantes que vivem nos Estados Unidos relatam discriminação racial por parte da polícia e da justiça (18, 42).

O estigma e os rígidos papéis de gênero influenciam o comportamento das mulheres, fazendo com que demorem a buscar ajuda (12, 16-18, 21, 26-28, 40, 42, 43, 45). Quando as mulheres buscam ajuda, tais desigualdades influenciam também as atitudes dos profissionais que as atendem, que por vezes maltratam as mulheres (32).

**TABELA 1:** Caracterização dos estudos sobre a temática da violência contra as mulheres, 2001 a 2017

Local da pesquisa	Delineamento metodológico	Principais achados	Referência
Grécia	Entrevistas em um abrigo para vítimas.	Demoram a buscar ajuda; buscam ajuda quando a violência se torna severa, falando com amigas, irmãs ou madrinhas.	11
Estados Unidos	Análise de formulários em um centro de atendimento para mulheres vítimas de violência doméstica.	Existem barreiras ambientais, culturais e sociais para a busca de ajuda.	12
Estados Unidos	Entrevistas para analisar a busca de ajuda.	Dificuldades econômico-culturais impedem a busca de ajuda. Primeiro procuram família e amigos.	13
Brasil	Entrevistas para identificar onde as mulheres buscam ajuda.	Buscam ajuda quando a violência é grave, recorrendo primeiro à família.	14
Estados Unidos	Entrevistas para verificar a ocorrência de violência e onde as mulheres buscam ajuda.	Quanto mais grave a violência, maior a procura por ajuda médica e jurídica.	15
Canadá	Grupos focais com imigrantes do Sul da Ásia.	Questões de gênero, apoio social pós-imigração e falta de conhecimento dificultam a busca por ajuda.	16
Canadá	Entrevistas com informantes chaves e grupos focais com imigrantes.	Não procuram apoio devido a barreiras legais e políticas.	17
Estados Unidos	Análise da busca de ajuda de imigrantes mexicanas.	Apoio informal associado a valores machistas e valorização familiar. Apoio formal associado a maior nível educacional.	18
Índia	Estudo descritivo sobre um centro de atendimento a mulheres vítimas de violência doméstica.	Recorrem a amigos, vizinhos, centros de referência e hospitais por iniciativa própria ou da comunidade.	19
Canadá	Inquérito telefônico e estudo transversal.	Conversam com família, amigos, colegas de trabalho, profissionais de saúde, advogado, padre, polícia e serviços sociais.	20
Sri Lanka	Estudo de caso.	Criação de um modelo teórico sobre as necessidades da busca por ajuda.	21
Canadá	Pesquisa sobre busca de ajuda formal e informal.	Quando a violência é mais severa, buscam mais apoio informal.	22
Índia	Análise da exposição à violência e busca de ajuda.	Procuram ajuda familiar e policial. As que trabalham buscaram mais ajuda.	23
Sérvia	Descritivo sobre o perfil de busca de ajuda.	22% das mulheres buscam ajuda formal relacionada ao sofrimento dos filhos.	24
Estados Unidos	Estudo descritivo com dados secundários.	Maior escolaridade resulta na procura de familiares, amigos, profissionais de saúde e polícia.	25
Brasil	Entrevistas sobre conhecimento da busca por ajuda.	Recorrem a família e amigos primeiramente, e depois a serviços de saúde e justiça.	26
Brasil	Entrevistas para estabelecer a rota crítica.	A rota de busca se baseia em amigos, colegas de trabalho, delegacias e profissionais de saúde.	27
Estados Unidos	Questionário e grupos focais sobre violência universitária.	Normas sociais do ambiente do câmpus influenciam a busca de ajuda.	28
Brasil	Análise de inquérito sobre violência doméstica.	Apoio informal para violência branda e apoio formal quando é mais grave.	29
Jordânia	Grupos focais com imigrantes para descrever suas experiências.	Percebem como apoio: família, abrigo, justiça, serviços de saúde, religião e líderes comunitários.	30
Turquia	Entrevistas sobre a violência e busca de ajuda.	A maioria buscou apoio informal. Buscam apoio formal quando não aguentam mais.	31
México	Pesquisa nacional sobre busca da justiça pelas mulheres quando sofrem violência.	26,08% buscaram ajuda da família, depois da polícia, autoridades públicas ou agências do governo.	32
Japão	Estudo de curso de vida.	Maior procura por apoio informal antes de recorrerem à ajuda formal.	33
Índia	Estudo transversal com casadas sobre rota de ajuda.	Conversaram com familiares, vizinhos, polícia, trabalhador social e ONG. Desconheciam locais de ajuda.	34
Paquistão	Entrevistas em um hospital sobre violência e busca de ajuda.	50% falaram com os pais, 48% não buscaram ajuda e 2% buscaram ajuda formal.	35
Estados Unidos	Entrevistas com imigrantes e informantes chaves.	Redes informais têm papel fundamental, inclusive na indicação para o apoio formal.	36
Nigéria	Transversal e descritivo.	A maioria não buscou ajuda. Quando procuraram, foi mais informal.	37
Brasil	Entrevistas em um Centro de Referência à Mulher.	O Centro de Referência foi procurado quando a violência alcançou o limite da tolerância.	38
México	Grupos focais para examinar o comportamento de busca de ajuda.	A maioria não recorreu à ajuda formal e declarou que a família não é fonte de apoio.	39
Estados Unidos	Entrevistas com mulheres sobre onde buscam ajuda.	Apoio formal relaciona-se com serviços jurídicos, abrigos e centro de referência, porém a maioria opta pelo apoio informal.	41
Estados Unidos	Grupos focais para descrever como as mulheres percebem situações violentas.	Fatores culturais e financeiros retardam a busca por ajuda. As participantes desconheciam os locais para de apoio formal.	42
Tanzânia	Grupos focais e entrevistas nas três regiões do país.	Falta de confiança na ajuda formal; normas sociais e de gênero dificultam a busca por ajuda.	40
Serra Leoa e Libéria	Entrevistas e grupos focais para verificar a busca de ajuda.	Apoio informal auxilia na tomada de decisão e a cultura tem influência.	43
Gana	Entrevistas semiestruturadas sobre rota de apoio.	Conheciam alguns serviços, mas desconheciam seu funcionamento. Nenhuma mulher conhecia a lei sobre violência doméstica.	44
Índia	Dados transversais sobre mulheres que sofreram violência física ou sexual.	Menos de um quarto procurou ajuda e apenas 1% em instituições formais.	45
Estados Unidos	Qualitativo, com uso de entrevistas sobre rota de ajuda.	28% das mulheres nunca buscaram apoio formal. As que procuravam eram em sua maioria negras.	46
Espanha	Entrevistas com imigrantes marroquinas, romenas e equatorianas.	84% buscaram ajuda formal, como serviço social, profissionais de saúde e polícia.	47
Tanzânia	Transversal para verificar a relação entre os recursos econômicos e suas respostas à violência.	40% procuraram ajuda em serviços de saúde, polícia e junto a líderes (religiosos e comunitários).	48

A maioria das mulheres que buscou ajuda formal (15, 20, 24, 29, 31-33, 35, 37, 39, 41) acreditava que a violência era culpa delas mesmas (24, 31, 32), pela existência de normas sociais que toleram a violência no âmbito do casamento, pela crença de que essas situações devem ser tratadas em casa (24, 28, 29, 32, 34, 40, 42), por acreditarem que a violência era suportável (20, 24, 29, 31, 32) ou que esse seria o último episódio (24), por não saberem a quem recorrer (20, 31, 34) e por terem medo das ameaças do parceiro (12, 29, 32).

A família aparece como principal recurso de apoio escolhido pelas mulheres e como fator impulsor na busca de ajuda formal, às vezes não configurando o suporte esperado (39). Ao buscarem seus pais, as mulheres encontraram neutralidade ou negatividade, principalmente por pressão relacionada à manutenção da família (11, 39, 42). Em certos casos, a família tentava persuadir as mulheres a não buscarem ajuda (32). Estudos apontam que, para evitar conflitos familiares, as mulheres optam pelo silêncio (27, 36, 42).

Não confiar nas instituições de atendimento apareceu como motivo para que as mulheres não buscassem ajuda nos Estados Unidos (12, 42), Tanzânia, Serra Leoa e Libéria (40, 43), Brasil (26, 27), México (32) e Índia (45). Outras barreiras que impediam a busca de ajuda foram falta de dinheiro (12, 13, 17, 40-43), pouco conhecimento e disponibilidade limitada de serviços de apoio (12, 16, 20, 21, 31, 48), por exemplo, centros de referência (12, 27), e falta de apoio/fragmentação da polícia/justiça/saúde (12, 21, 27, 43).

### Rota crítica das mulheres no mundo

As mulheres podem levar anos até decidirem buscar ajuda, dadas as diversas barreiras já abordadas neste artigo. O primeiro passo para a busca de ajuda é reconhecer que vivenciam situação de violência.

Identificou-se que o sistema de apoio informal escolhido pela maioria das mulheres compreende família e amigos (10-12, 15, 17, 20, 22-27, 29, 30-35, 37, 38, 43, 44, 46-48). Entre os membros da família, a mãe e a irmã são as primeiras opções, seguidas dos irmãos e, por último, do pai (11, 24, 31, 36).

Quanto maior a influência das questões sociais de valorização da família e do gênero, mais as mulheres procuram a própria família ao invés de instituições formais (18). Também buscam apoio familiar quando não consideram a violência grave (14, 31). A família se mostrou fundamental na busca por apoio formal, indicando serviços onde as mulheres poderiam ser auxiliadas (13, 19, 36).

Apesar de o agressor ser, geralmente, o companheiro/marido, a família do parceiro foi citada nos artigos como importante recurso de busca de ajuda (14, 23, 25, 31, 34, 36, 43). Na família do companheiro/marido, buscam-se principalmente as mulheres (31) e o sogro (31, 36). Geralmente, os parentes do cônjuge tentam mediar a situação de conflito, apoiando para que o casal permaneça junto (43).

Algumas vezes, os vizinhos estão entre os primeiros a saber sobre a violência (19, 29, 31, 34, 43), portanto tornam-se um importante recurso de ajuda (43). Muitas mulheres ficaram sabendo da existência de centros de referência para atendimento e aconselhamento por meio de vizinhos (19).

A busca por líderes religiosos, como padres e pastores (14, 20, 23, 29, 30, 37, 46, 48), pode ser mais frequente do que a busca por locais de atendimento formais (14). Entretanto, em estudos realizados nos Estados Unidos (12, 13, 15, 18, 25, 28, 36, 41, 42, 46) e países da Europa (24, 11, 47), os líderes religiosos não são citados como rede de apoio, apontando para a necessidade de estudos com foco na influência da religião nas questões de violência doméstica em diferentes países.

Em alguns casos, a religião configura-se como justificativa para a violência, a partir de interpretações incorretas de livros sagrados (Alcorão), ou contribui para a naturalização da violência contra a mulher a partir da ideia de subserviência feminina (30, 45). Tais valores estão estruturados por meio da subjetividade e do plano simbólico, embasando comportamentos sociais que podem contribuir para a manutenção de uma relação desigual e violenta (19).

No que se refere ao sistema formal, identificou-se que os setores policial e da saúde são os mais procurados segundo os artigos analisados, seguidos pelos serviços sociais (como abrigos), sistema de justiça e advogados e por último as ONG. A polícia aparece como o local mais citado (12-14, 16, 18, 20, 24, 25, 27, 29, 31, 32, 34, 35, 38, 39, 42, 46, 47), sendo acionada principalmente quando a violência é severa (27, 29, 35, 42) pelas mulheres mais jovens. As mais jovens buscam mais a polícia do que locais de atendimento para a saúde (47), mesmo estando pouco satisfeitas com o serviço policial, por sentirem-se seguras (25, 47).

Os serviços de saúde também aparecem com frequência. Os principais serviços procurados são hospitais, unidades de atenção primária e ambulatórios de saúde mental (16, 19, 20, 22, 26, 28, 29, 31, 34, 35, 37, 38, 44, 47, 48). São citados profissionais como médicos, enfermeiros, psiquiatras, psicólogos, dentistas ou outros profissionais de saúde (13, 17, 23, 36, 44). A maioria das mulheres visitou uma unidade de saúde no último ano (13), às vezes porque a violência sofrida foi tão grave que necessitou de cuidados de saúde (46). Além disso, os serviços de saúde foram as instituições com maior potencial para ajudar as mulheres, segundo a avaliação delas mesmas (19, 26).

Os abrigos de proteção foram procurados para suporte devido às dificuldades financeiras e necessidade de auxílio com os filhos, à gravidade da violência (15, 16, 25, 32, 44, 47) e também quando havia muito controle por parte do agressor (36). Os centros de referência foram citados como locais onde as mulheres buscam apoio (17, 21, 22, 27, 28, 32, 33, 44) principalmente com violência grave (29, 36). Um dos artigos se refere à pesquisa realizada com mulheres atendidas em um centro de referência (32). Outro fator importante relativo aos centros de referência é a sua escassez, que é uma das barreiras encontradas para a busca de ajuda formal (19, 32).

Na busca por ajuda, algumas mulheres optaram por acionar o sistema de justiça e advogados (16, 17, 19, 25, 36, 44), principalmente nas formas graves de violência e na violência de longa data (34). Os operadores jurídicos, algumas vezes, pressionaram as agredidas a permanecerem na relação conjugal (46). As ONG aparecem como recurso na minoria dos artigos analisados (31, 34, 35, 43, 44) e aparecem como local buscado pelas mulheres que vivem em países da África (43, 44) e Ásia (31, 34, 35).

## DISCUSSÃO

A compreensão do fenômeno da violência contra a mulher através da rota crítica aponta caminhos possíveis para o enfrentamento do problema, bem como fatores que podem predispor ou limitar a vítima na busca por apoio. A escolaridade e a renda da mulher, observadas em estudos nessa revisão, têm sido discutidas pela OMS. Essa organização tem recomendado estratégias para aumentar o empoderamento econômico e social das mulheres, no intuito de garantir a redução da violência doméstica (49). Além disso, sendo apontada como um fator de risco para a violência praticada pelo parceiro, a baixa escolaridade também deve ser enfrentada, com fortalecimento de ações ligadas à ampliação do acesso à educação para as mulheres e seus parceiros, sobretudo os de baixa renda (49). Espera-se que uma maior escolaridade favoreça um melhor entendimento sobre leis e políticas contra a violência, além do reconhecimento de sinais violentos por parte da vítima, o que pode favorecer a redução do fenômeno.

Em relação aos aspectos sociais e culturais, vários dos motivos que limitam a busca de ajuda estão relacionados com barreiras culturais decorrentes das normas de gênero e convenções sociais tradicionais, como as ligadas à família, que toleram a violência e a desigualdade entre homens e mulheres em seus papéis sociais. Observa-se, por exemplo, que alguns países africanos ainda têm práticas tradicionais nocivas, como a mutilação genital feminina (50). Na Jordânia e Índia o casamento é considerado sagrado e a violência contra a esposa é permitida em alguns casos, como na presença de infidelidade (30, 34). Pondera-se aqui que as barreiras culturais estão presentes na maioria dos estudos incluídos nesta revisão, independentemente da condição econômica do país estudado. Observa-se, portanto, que as desigualdades de gênero estão inseridas na realidade social de países desenvolvidos e em desenvolvimento. Assim, é preciso romper com a visão estereotipada de que apenas em locais de baixa renda as normas de gênero levam a iniquidades e favorecem a violência contra as mulheres.

Os contextos de imigração, onde as condições de deslocamento expõem diferenças culturais importantes entre grupos sociais, foram observados nesta revisão como fatores inibidores para procura por ajuda. Debates sobre os processos de inclusão de imigrantes e refugiadas nas redes de apoio formais para superação da violência doméstica ainda são incipientes, mesmo com o crescimento recente da discussão a respeito da questão migratória mundial (51). É possível afirmar que as violações preponderantes entre as mulheres imigrantes se dão, sobretudo, por ainda existirem desafios na promoção da autonomia dessas mulheres.

Mesmo que no início deste século (ano de 2000), a Organização das Nações Unidas (ONU) tenha sintetizado diversos acordos internacionais com a Declaração do Milênio (52), dentre eles a promoção da igualdade entre os sexos e a autonomia das mulheres, ainda existe um longo caminho a ser percorrido para reduzir a violência contra a mulher em todo o mundo. As normas patriarcais e de domínio masculino refletem a desigualdade, produzem iniquidades em nível social e validam a violência praticada pelo parceiro íntimo (48). As normas de gênero e as questões decorrentes desse acordo social são frequentes em diversas sociedades; os pontos em comum se referem a um papel subserviente da mulher.

As dificuldades de buscar ajuda por motivos internos e individuais, bem como por motivos externos, pautados por barreiras socioculturais, como o familismo, normas rígidas de gênero, patriarcado e dependência econômica do parceiro devem ser levadas em consideração na proposição de estratégias para a redução de práticas violentas contra a mulher. Nos estudos verificados, a busca por apoio formal em situações de violência doméstica é precedida pelo apoio informal, com destaque para família, vizinhos e líderes religiosos. Assim, a indicação de possíveis locais de ajuda é, frequentemente, feita por pessoas próximas da vítima, reforçando-se a importância do nível social, do modelo ecológico de violência adotado pela OMS e das normas rígidas de gênero nos níveis comunitários relacional e individual (49).

Esta revisão identificou que a interferência da comunidade e da rede de apoio pode ser fator protetor ou de risco, dada a complexidade de aspectos inseridos no ciclo da violência alinhado ao apontado pela OMS (49). Para garantir apoio na busca de ajuda, com rompimento no ciclo de violência, é preciso trabalhar com a sociedade o conceito de gênero e sua influência nas atitudes violentas (41, 43), bem como o conceito e a definição de violência e os recursos disponíveis para seu enfrentamento.

Destaca-se a importância de que discussões relacionadas ao tema sejam ampliadas para a sociedade, em âmbito familiar e comunitário, inclusive com líderes religiosos, já que muitas mulheres buscam compreender a relação de violência que sofrem por meio da religião; esta também pode influenciar parceiros abusivos a repensarem atos violentos no ambiente doméstico (14).

Entre os serviços que compõem a rede de apoio formal, o destaque para os serviços de saúde e para a polícia levantam questões sobre a necessidade de qualificar os processos de trabalho nesses espaços. Vale notar ainda a importância dos profissionais estarem sensibilizados e capacitados para trabalhar com situações de violência doméstica.

Algumas características são importantes para que as mulheres falem sobre a situação que vivenciam, como atitudes de não julgamento, ouvir amigavelmente e ter escuta que respeita as diferentes culturas (13, 22, 29, 49). A polícia, nesse contexto, desempenha ainda um papel crucial na rota crítica, favorecendo desfechos positivos ou negativos na superação da problemática.

Os centros de referência à vítima foram também citados como locais importantes nas rotas das mulheres. Recomenda-se que sejam ampliados para se tornarem mais acessíveis à população. As ONG, por outro lado, foram pouco discutidas no contexto de superação da violência. Sugere-se, portanto, novas pesquisas que abordem tais espaços de auxílio, o que poderia contribuir com melhoria e ampliação da rede de atenção.

Esta revisão contribui com a produção de conhecimento sobre a temática que envolve a rota crítica. São limitações deste estudo a inclusão apenas de artigos completos disponíveis em bases dados, não tendo sido consultados outros materiais, o que não permitiu, entre outros, análises de pesquisas anteriores a 2001. Ainda, não foram encontrados estudos sobre a Oceania, o que não proporcionou análise global, e não foram analisadas publicações em espanhol nem em outras línguas exceto inglês e português, não permitindo uma comparação entre diferentes países em cada continente. A influência dos desencadeadores da violência de acordo com cada cultura merece análises mais aprofundadas, tendo em vista sua importância e magnitude para proposição de intervenções efetivas na rota crítica das mulheres em situação de violência.

Em conclusão, a rota crítica das mulheres em situação de violência doméstica no mundo existe de maneira formal e informal. Inicia, principalmente, de maneira informal, com família, amigos, vizinhos e líderes religiosos. As mulheres buscam ajuda formal conforme aumenta a gravidade e a severidade dos casos, junto a polícia, serviços de saúde, locais de apoio social, justiça/advogados e ONG. Os principais fatores que impulsionam a busca de ajuda são empoderamento econômico e maior escolaridade. Já como fatores inibidores, destacam-se o *status* de imigrante, motivos internos e individuais e presença de barreiras institucionais e socioculturais, como cultura patriarcal, normas rígidas de gênero e dependência financeira.

## Contribuições dos autores.

DYB e EMS elaboraram o projeto de pesquisa e redigiram a primeira versão do artigo. DYB, ACAR, CPC e MCM realizaram a coleta de dados e a redação final do artigo. Todos os autores revisaram e aprovaram a versão definitiva.

## Declaração.

As opiniões expressas no manuscrito são de responsabilidade exclusiva dos autores e não refletem necessariamente a opinião ou política da RPSP/PAJPH ou da Organização Pan-Americana da Saúde (OPAS).
